# Topical Treatment of Colquhounia Root Relieves Skin Inflammation and Itch in Imiquimod-Induced Psoriasiform Dermatitis in Mice

**DOI:** 10.1155/2022/5782922

**Published:** 2022-01-11

**Authors:** Fei Li, Dan Han, Bo Wang, Wentao Zhang, Yan Zhao, Jing Xu, Liesu Meng, Kuanhou Mou, Shemin Lu, Wenhua Zhu, Yan Zhou

**Affiliations:** ^1^Department of Dermatology, The First Affiliated Hospital of Xi'an Jiaotong University, Xi'an, Shaanxi, China; ^2^Center for Translational Medicine, The First Affiliated Hospital of Xi'an Jiaotong University, Xi'an, Shaanxi, China; ^3^Institute of Molecular and Translational Medicine, and Department of Biochemistry and Molecular Biology, School of Basic Medical Sciences, Xi'an Jiaotong University Health Science Center, Xi'an, Shaanxi, China; ^4^Key Laboratory of Environment and Genes Related to Diseases (Xi'an Jiaotong University), Ministry of Education, Xi'an, Shaanxi, China

## Abstract

Itch is one of the major clinical manifestations of psoriasis, which is closely related with neurogenic inflammation and difficult to control. Colquhounia Root (CR) is a Chinese herb exhibiting broad bioactivities on anti-inflammation. This study was designed to explore the antipsoriatic and anti-itch potential of CR and its underlying mechanisms. Mice in a model of imiquimod-induced psoriasiform dermatitis were treated topically with CR for 7 days, and the severity of skin lesions and itch was significantly ameliorated. CR reduced the inflammatory cell infiltration, as well as mast cells in skins. Particularly, the expression of inflammatory cytokines and chemokine including *Il17a*, *Il22*, and *Ccl20* and itch-related molecules such as *SP*, *CGRP*, and *NGF* in lesions were decreased in diseased mice upon application with CR. The normal human epidermal keratinocytes were stimulated with the M5 cytokine cocktail, the mixture of IL-17A, IL-22, Oncostatin M, IL-1*α*, and TNF-*α*, and cell viability and mRNA expression levels of inflammatory factors and itch-related molecules were measured after being treated with CR. We found that CR inhibited both cell hyperproliferation and overexpression of inflammatory cytokines and itch-related molecules *in vitro*. Altogether, we conclude that CR relieves psoriatic lesions and itch via controlling immunological and neurogenic inflammation.

## 1. Introduction

Psoriasis is a chronic immune-mediated inflammatory disease mainly manifested as scaly erythema and patches in the skin. Patients suffering from psoriasis often complain of disgusting appearance, chronic itch, and joint pain [1–3]. Itch is one of the most troublesome symptoms of psoriasis, affecting the quality of life and sleep of patients. It is probably unrelated to the severity of psoriasis due to its particular mechanisms [4].

The pathophysiological mechanism of itch in psoriasis, which is mainly related to mental stress and neurogenic inflammation, remains poorly understood. Studies have shown that itch-related molecules released by epidermal keratinocytes and other cells in the dermis such as calcitonin gene-related peptide (CGRP), substance P (SP), nerve growth factor (NGF), and TRPV1 and inflammatory cytokines (IL-17 and IL-22), as well as chemokine CCL20, are important in mechanisms of itch [5–10]. Their expressions are significantly increased in the skin specimens of psoriasis patients, involved in the occurrence and development of psoriatic itch [2, 11].

In the last few decades, considerable progress has been made in the treatment of psoriasis including topical or oral traditional medications, novel biologics, and phototherapies. With the in-depth study of psoriasis, psoriatic itch has gradually been noticed. However, traditional antipruritic therapies (such as antihistamines) are limited in the treatment of itch in psoriasis. Traditional topical drugs like vitamin D3 derivatives and coal tar for clinical treatment of psoriasis have little effect on itch or even aggravate it [12]. Biological agents, such as TNF-*α* and IL-17 inhibitors, can quickly and effectively relieve itch, but some patients could not afford them because of high prices or high-risk adverse reactions [13], such as infection, urticaria, and tumor in the long term [14]. Therefore, successful treatment of psoriatic itch is still challenging. It is necessary to further explore and develop treatment strategies and drugs to relieve psoriatic itch.

Nowadays, the use of botanical therapeutics has been taken attention by dermatologists, such as Tripterygium wilfordii and Colquhounia Root (CR) [15]. CR is a traditional Chinese medicine, containing several bioactive ingredients such as triptolide and tripterine [16], is widely used in the treatment of autoimmune-related diseases such as systemic lupus erythematosus, rheumatoid arthritis, chronic nephritis, vasculitis, and psoriasis, with anti-inflammatory and analgesic effects [17–19]. However, it has not been reported whether CR has the effect of inhibiting itch in psoriasis. Therefore, we aim to explore the effects and mechanisms of topical treatment with CR.

## 2. Materials and Methods

### 2.1. Mice

Forty-one female C57BL/6J mice, weighing 18-20 g, were purchased from Xi'an Jiaotong University Laboratory Animal Center and were kept at a standardized breeding environment. Standard diet and water were available to all mice anytime. All animal experiments were approved by the Institutional Animal Ethics Committee of Xi'an Jiaotong University.

### 2.2. Preparation of CR

CR tablets (Z20027411) were provided by Pharmaceutical Factory of the Chongqing Academy of Chinese Mataria Medica. We grounded 5 g CR tablets into powder and then mixed it in 250 g Vaseline to keep the effective concentration which was 0.002%, and then stored it at 4°C.

### 2.3. Imiquimod- (IMQ-) Induced Psoriasiform Dermatitis (PsD) in Mice and Treatment

**Figure 1:**
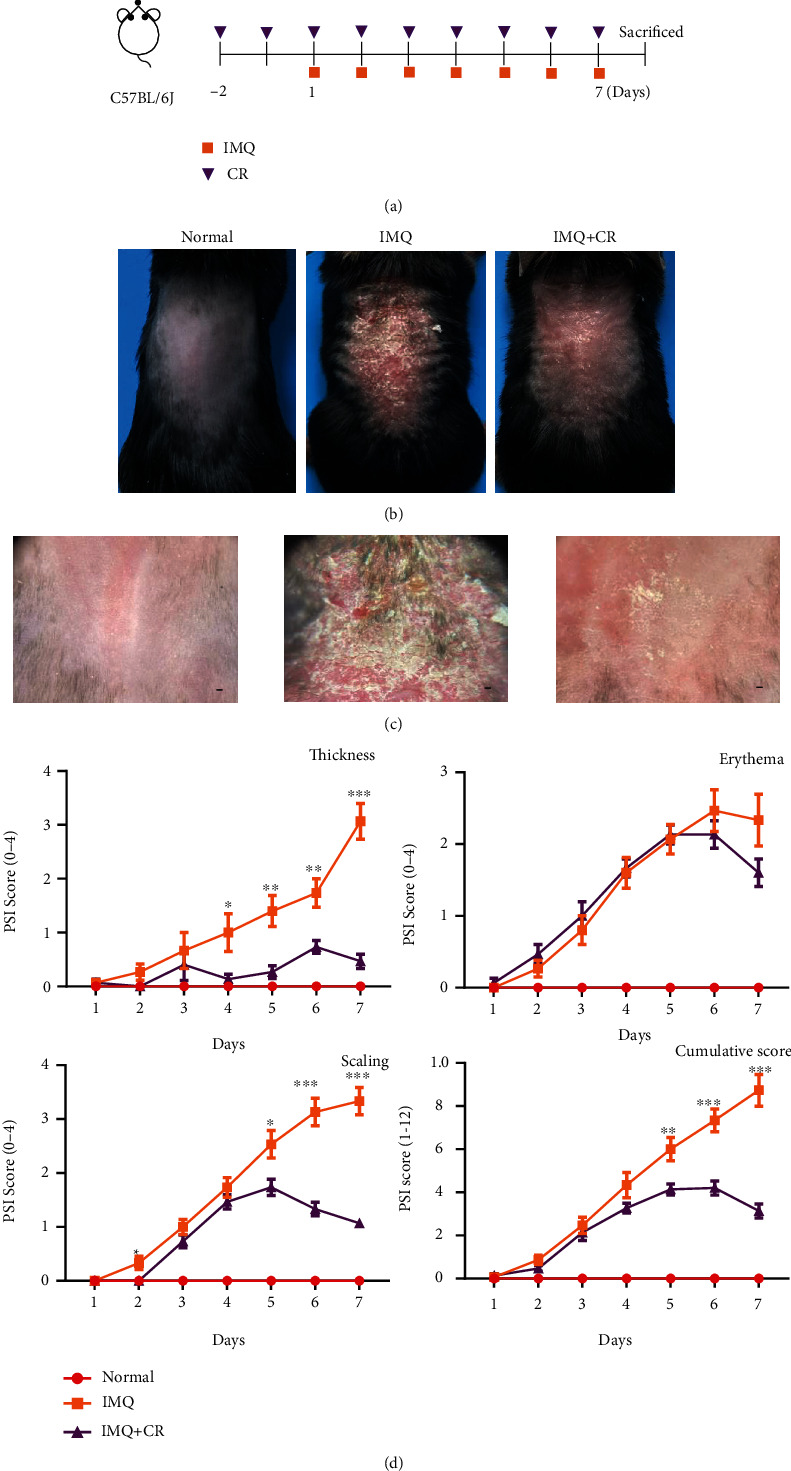
CR treatment attenuated the severity of IMQ-induced PsD in mice. (a) The scheme of IMQ-induced murine model and CR treatment. Clinical presentation of mice (b) and dermoscopic presentation (scale bar = 1 mm) (c) of the back skin of mice after 7 days of IMQ treatment with or without the topical application CR. (d) The daily scores of erythema, skin thickness, scaling of back skin, and PSI scores were compared among normal group (*n* = 11), IMQ group (*n* = 15), and IMQ + CR group (*n* = 15). Data are shown as the Mean mean ± SEM and were analyzed by using *t*-test. ^∗^*P* < 0.05,  ^∗∗^*P* < 0.01, and^∗∗∗^*P* < 0.001, showing the difference between the IMQ group and IMQ+CR group.

Mice were randomly divided into three groups, the normal control group, disease group (IMQ), and CR treated group (IMQ+CR). We followed the methods of William et al. to establish the IMQ-induced psoriasiform dermatitis mouse model [20]. Mice were anesthetized with isoflurane, and their hair on the back skin was shaved (3 cm × 3 cm). Then, 62.5 mg of IMQ creams (Mingxin Pharmaceutical Co., Ltd., Sichuan, China) was applied daily onto the dorsal skins for 7 consecutive days to induce psoriasiform dermatitis in the IMQ group and IMQ+CR group mice. CR were daily administered to the dorsal skins for 9 consecutive days from two days before IMQ application. Mice in the normal group were treated with Vaseline. A blinded observer measured the thickness of the dorsal skins by a vernier caliper daily and observed and photographed the back skin with a dermoscope simultaneously. Psoriasis Severity Index (PSI) scores were used to evaluate the severity of skin lesions every day [21], which were graded from 0 to 4 for erythema, thickness, scaling, and total. All mice were euthanized, and their skin specimens were stored for determination and further experiments. The experiment scheme is shown in Figure 1(a). Liver and renal injury biomarkers were carried out by an autoanalyzer (LABOSPECT 008 ASi, Hitachi, Japan).

### 2.4. Behavioral Tests

We did the behavioral tests following the methods of Sakai et al. [22]. Firstly, we pretreated and habituated all mice to a transparent acrylic box for 30 min twice. Twenty hours after each topical application, we videotaped the number of scratch bouts of mice for 30 minutes. Then, the scratch bouts of mice in videos were counted by a blinded observer. A scratch bout was defined as once or more times rapidly front and backward pointing and touching movement with hind claws in the skin lesions and ending by licking or biting the toes or placing the back claws on the floor. Other movements away from the treated area as ear scratching and grooming were excluded.

### 2.5. Histopathology and Immunohistochemistry (IHC)

Skin specimens in paraformaldehyde-fixed (4%) were embedded in paraffin for haematoxylin and eosin staining (H&E) and IHC. Mast cells were stained by Toluidine blue following the way of Puebla-Osorio et al. [23]. We assessed the histopathological alterations with computer-assisted quantitative image analysis in lesions, including epidermal thickness, area of Munro's microabscesses (MM), and the number of mast cells. Tissue sections were prepared for IHC with SP (Cat, No. sc-21715, 1 : 50, Santa Cruze), CGRP (Cat, No. sc-57053, 1 : 50, Santa Cruze), and NGF (Cat, No. sc-32300, 1 : 50, Santa Cruze) antibodies. The integrated optical density (IOD) of SP, CGRP, and NGF in the image was measured by Image Pro plus 6 software.

### 2.6. mRNA Quantification

RNA, extracted from skin specimens with TRIzol reagent (Thermo Fisher Scientific) according to the instruction, was converted into cDNA with the RevertAid First Strand cDNA Synthesis kit (Thermo Fisher Scientific). Quantitative real-time polymerase chain reaction (qPCR) was performed on a PCR machine (CFX CNNNECT Real-time system; Bio Rad, Hercules, CA, USA). The gene-specific primers are summarized in Table 1. The data were analyzed with the 2^−*ΔΔ*CT^ method and normalized by GAPDH.

### 2.7. Flow Cytometry

Skin samples from each group were cut into pieces using ophthalmic scissors and digested using collagenases (2.6 mg/ml, Sigma) and DNase (0.1 mg/ml, Sigma) for 1.5 h. The cell suspension was filtered by a 40 *μ*m nylon net. Then, cells from the skin were stained with anti-mouse-BV785-CD45.2 (Cat, No. 109839, 1 : 200, BioLegend), anti-mouse-AF700-TCR*β* (Cat, No. 109224, 1 : 200, BioLegend), anti-mouse-PE-Cy5-CD4 (Cat, No. 100514, 1 : 200, BioLegend), anti-mouse-BV711-CD8 (Cat, No. 100759, 1 : 200, BioLegend), anti-mouse-APC-CD11b (Cat, No. 101212, 1 : 200, BioLegend), anti-mouse-FITC-F4/80 (Cat, No. 123108, 1 : 200, BioLegend), anti-mouse-Pacific blue-Ly6G (Cat, No. 127612, 1 : 200, BioLegend), and anti-mouse-PE-*γδ*T (Cat, No. 553178, 1 : 200, Becton Dickinson). Finally, we detected and analyzed all samples with a flow cytometer. Gating strategy of cells in flow cytometry is shown in supplementary Figure 1.

### 2.8. Cell Culture and Treatment

We performed all culture experiments on the normal human epidermal keratinocytes (NHEKs) which were obtained from ATCC (Manassas, VA). Cells were cultured with Dulbecco's modified Eagle's medium (DMEM, Hyclone) supplemented with 10% heat-inactivated fetal bovine serum (FBS), 100 U/ml of penicillin, and 100 *μ*g/ml of streptomycin and were placed in an incubator at a 37°C humidified atmosphere with 5% CO_2_. NHEKs were treated with 10 ng/ml of M5 cocktail (IL-17A, IL-22, Oncostatin M, IL-1*α*, and TNF-*α*) [24], with or without CR dissolved in DMSO for 24 h.

### 2.9. Cell Viability Assay

We tested the effect of CR on the growth of NHEKs by CCK-8 assay following the methods of Ru et al. 2020 [25]. We seeded NHEKs onto 96-well plates at a density of 2000 cells per well. Then, the medium was replaced with fresh medium containing 10 ng/ml M5 and various concentrations (0, 0.25 *μ*g/ml, 0.5 *μ*g/ml, 1 *μ*g/ml, and 2 *μ*g/ml) of CR and medium containing 0.1% DMSO were used as the vehicle control after 24 h. After 24 h incubation, 10 *μ*l of CCK8 solution (Cat. No. AR1160, Boster) was added to each well. Following 4 h incubation at 37°C, the absorbance of each well at 450 nm was determined.

### 2.10. Statistical Analysis

Results are expressed as the mean ± SEM and were analyzed statistically using Graphpad Prism software. A *t*-test was used to compare the differences between two groups. One-way ANOVA with a post hoc comparison (Tukey's HSD) test was used to compare the differences in more than two groups. Statistical significance was defined as *P* < 0.05.

## 3. Results

### 3.1. Topical Treatment of CR Alleviates the Psoriasis-Like Lesions Induced by IMQ

The IMQ-induced PsD model is one of the best psoriatic mouse models with similar immunological alteration to human [26]. In order to understand the anti-inflammatory effects of CR, we applied CR to IMQ-induced mice. Topical treatment of CR has shown no obvious damage to liver and renal function of mice or even improved related index compared with the IMQ group (Supplementary figure 2).

The erythema, thickness, and scaling were observed after IMQ application (Figures 1(b) and 1(c)). The severity of these lesions was increased continuously with daily IMQ application (Figure 1(d)). By comparison, the severity of the skin lesions observed in the IMQ-induced PsD mice was reduced by the coadministration of CR (Figures 1(b) and 1(c)). In these mice, reduced scales, skin thickness, and cumulative scores were observed from day 5 to day 7 compared with mice in the model group (Figure 1(d)). However, the erythema of skin in the CR-treated group was not relieved (Figure 1(d)). Under the dermoscope, regularly distributed dotted vessels and scales were observed in the IMQ group, while they were diminished in the CR-treated group.

**Figure 2:**
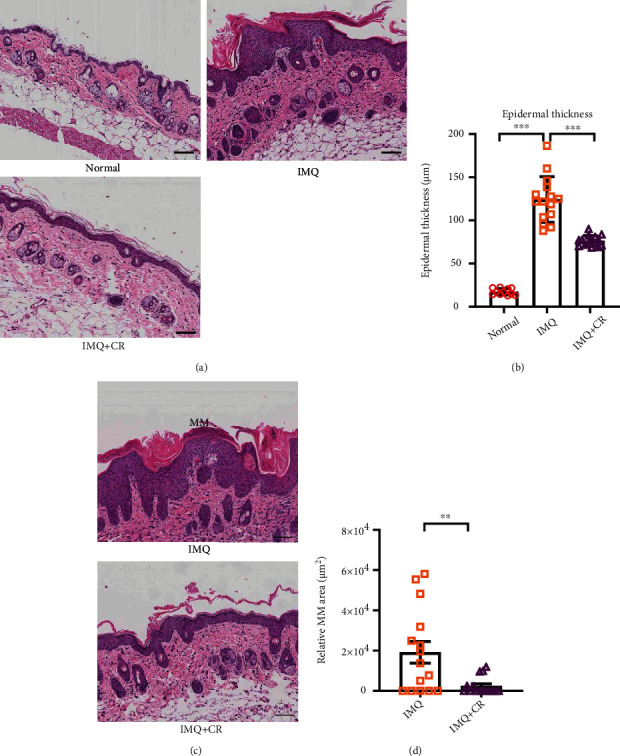
CR treatment reduced the epidermal thickness and diminished neutrophil accumulation in IMQ-induced PsD in mice. (a) Representative microscopic images of H&E-stained skin sections from each group (scale bar = 145 *μ*m). (b) Epidermal thickness was evaluated using Leica Microsystems software under a microscope. *n* = 11 in the normal group; *n* = 15 in IMQ group and IMQ+CR group. (c) Representative image of MM (scale bar = 145 *μ*m). (d) Quantification of MM area. *n* = 15 in both the IMQ and IMQ+CR group. Epidermal thicknesses were compared by using one-way ANOVA and post hoc Tukey's test, and MM areas were compared using a *t*-test. Data are shown as the Mean ± SEM. ^∗∗^*P* < 0.01 and^∗∗∗^*P* < 0.001.

The sections of the IMQ-treated group showed the representative histopathological changes of psoriasis with hyperkeratosis, parakeratosis, and acanthosis, while these features were absent in normal mice (Figure 2(a)). However, CR treatment partially inhibited these characteristic changes in psoriatic lesions (Figure 2(a)). Statistical analysis also showed a significant decrease in the epidermal thickness in CR-treated mice (Figure 2(b)). In human psoriasis, neutrophils aggregation in the cornified layer known as MM, indicated that neutrophils may play an important role in disease pathogenesis [27]. IMQ-treated skins also showed MM formation (Figure 2(c)). MM in the model group were larger and more numerous than those observed in CR-treated mice (Figure 2(c)). The average area of total MM of each mouse was dramatically diminished in CR-treated mice vs. model mice (*P* < 0.01) (Figure 2(d)).

Taken together, these results suggest that CR treatment could alleviate the severity of IMQ-induced PsD in mice. Therefore, it is necessary to study how this treatment exerts its therapeutic effect on psoriasis.

### 3.2. CR Treatment Improves the Immune Microenvironment of the Psoriatic Skins of Mice

**Figure 3:**
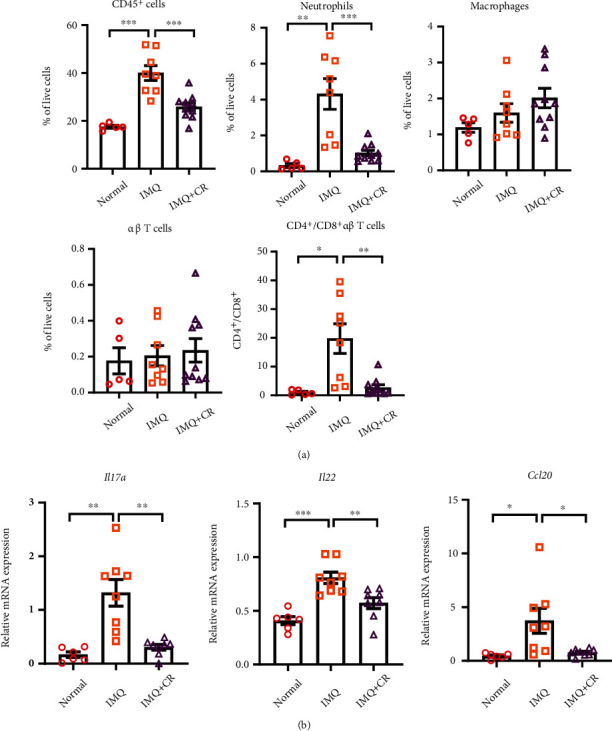
CR treatment decreased immune cell infiltration and cytokine production in murine skins of IMQ-induced PsD. (a) Flow cytometry analysis of immune cells (CD45^+^ immune cells, neutrophils, macrophages, *αβ* T cells, and CD4^+^/CD8^+^*αβ* T cells) from skin tissues among groups. *n* = 5 in the normal group, *n* = 8 in the IMQ group, and *n* = 10 in the IMQ+CR group. (b) mRNA expression of *Il17a*, *Il22*, and *Ccl20* in mouse skin was measured via RT-qPCR, and the relative mRNA expression was normalized to *Gapdh* expression. *n* = 6 in the normal group, *n* = 8 in the IMQ group, and *n* = 8 in the IMQ+CR group. Statistical analysis was performed using one-way ANOVA followed by post hoc Tukey's test. Data are shown as the Mean ± SEM. ^∗^*P* < 0.05,  ^∗∗^*P* < 0.01, and^∗∗∗^*P* < 0.001.

The immune cell infiltration in mouse skins was then evaluated by flow cytometry. After administration of IMQ cream for 7 days, a significant increase of CD45^+^ cells ratio was observed in the IMQ group (*P* < 0.001) compared with the normal group (Figure 3(a)). The ratio of neutrophils located in the skins was also increased after applying IMQ (*P* < 0.01) (Figure 3(a)). CR treatment significantly decreased both CD45^+^ cells and neutrophils (*P* < 0.01). However, the ratio of macrophages and *αβ*T cells in the skin showed no statistical differences between each group, whereas the proportion of CD4^+^ and CD8^+^*αβ*T cells was increased in the IMQ model group and decreased in the CR treatment group (Figure 3(a)).

Th17-related cells play an important role in inducing dermal inflammation and epidermal hyperplasia in psoriasis [28], so we analyzed the expression levels of inflammatory cytokines and chemokines in the mouse skins. IMQ treatment significantly induced the expression of *Il17a*, *Il22*, and *Ccl20* compared with the normal group, which showed the proinflammatory effect of IMQ by promoting the release of inflammatory factors. Moreover, a significant decrease in the levels of *Il17a*, *Il22*, and *Ccl20* was observed after CR treatment (Figure 3(b)).

These results suggest that topical CR treatment is more likely to have a direct anti-inflammatory effect on IMQ-induced PsD in mice.

### 3.3. Topical CR Application Relieves Psoriatic Itch and Reduces Itch-Related Mediators of Skins

**Figure 4:**
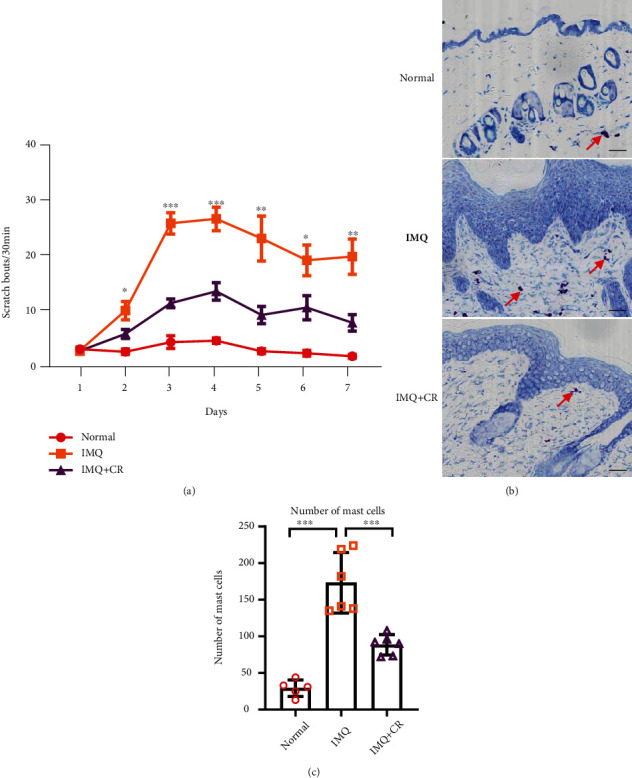
Pruritus was improved after CR treatment in IMQ-induced PsD in mice. (a) Spontaneous scratching was measured and compared among groups. *n* = 11 in the normal group and *n* = 15 in both the IMQ group and IMQ+CR group. (b) Mast cells were stained using Toluidine blue (scale bar = 73 *μ*m). (c) Numbers of mast cells in each group were counted. *n* = 5 in the normal group and *n* = 6 in both the IMQ group and IMQ+CR group. Statistical analysis was performed using one-way ANOVA followed by post hoc Tukey's test. Data are shown as the Mean ± SEM. ^∗^*P* < 0.05,  ^∗∗^*P* < 0.01, and^∗∗∗^*P* < 0.001.

As the IMQ mouse model is widely used in itch research of psoriasis, we observed the CR's effect on mice's itch behavior [22]. The counts of spontaneous scratch bouts were gradually increased in IMQ-treated mice compared with the control group (Figure 4(a)). CR application significantly reduced the scratch bouts since day 2 to day 7 (Figure 4(a)) and also diminished mast cells in dermis (Figures 4(b) and 4(c)). Mast cells are found to play a role in the induction or aggravation of psoriatic itch in previous studies [29]. The number of mast cells in the IMQ group was significantly higher than that in the normal control group. The reduction of mast cells by CR treatment was consistent with the results of itch behavioral test (Figures 4(b) and 4(c)). Both results suggest that CR can alleviate itch in psoriatic mice.

**Figure 5:**
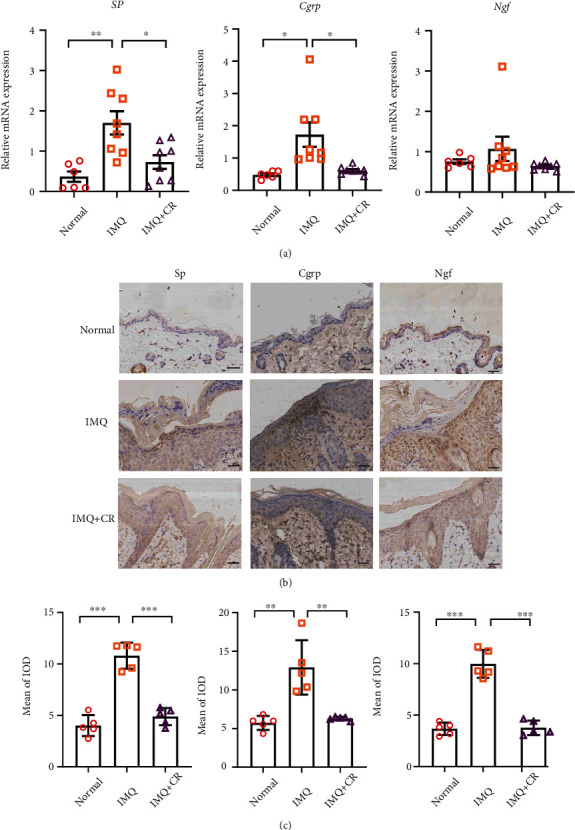
CR treatment reduced the expression of itch-related molecules in IMQ-induced PsD in mice. (a) mRNA expression of *Sp*, *Cgrp*, and *Ngf* in skins was detected by RT-qPCR. *n* = 6 mice in the control group and *n* = 8 mice in both the IMQ group and IMQ+CR group. Results of mRNA were normalized to *Gapdh* expression. (b) Representative immunohistochemical images of SP, CGRP, and NGF expression from the epidermis of back skins among groups (scale bar = 73 *μ*m). (c) Mean of integrated optical density (IOD) of Sp, Cgrp, and Ngf in skins from each group. *n* = 5 mice in three groups. Statistical analysis was performed using one-way ANOVA followed by post hoc Tukey's test. Mean ± SEM values are indicated, ^∗^*P* < 0.05,  ^∗∗^*P* < 0.01, and^∗∗∗^*P* < 0.001.

The pathogenesis of itch in psoriasis is still not fully understood. Nowadays, it is reported to be closely related with neurogenic inflammation [2]. To determine how CR treatment regulated itch in the IMQ model, we measured mRNA expression of itch-related genes *Sp*, *Cgrp*, and *Ngf* by RT-qPCR in skins. *Sp* (*P* < 0.01) and *Cgrp* (*P* < 0.05) in the IMQ group were increased vs. those in the control group (Figure 5(a)). Of note, *Sp* (*P* < 0.05) and *Cgrp* (*P* < 0.05) in the CR group were both reduced compared with those in the IMQ group (Figure 5(a)). But there were no differences in mRNA levels of *Ngf* among the three groups (*P* > 0.05) (Figure 5(a)). The same results were also confirmed from protein level by IHC. The Sp, Cgrp, and Ngf in the epidermis of psoriatic lesions were all increased markedly (*P* < 0.01) with IMQ application compared with the normal group, which were reduced (*P* < 0.01) after CR treatment (Figures 5(b) and 5(c)). Thus, it suggests that CR could negatively regulate itch-related molecules in PsD mice's skin to control itch.

### 3.4. CR Downregulates M5-Induced Inflammatory Cytokines and Itch-Related Molecules in NHEKs

**Figure 6:**
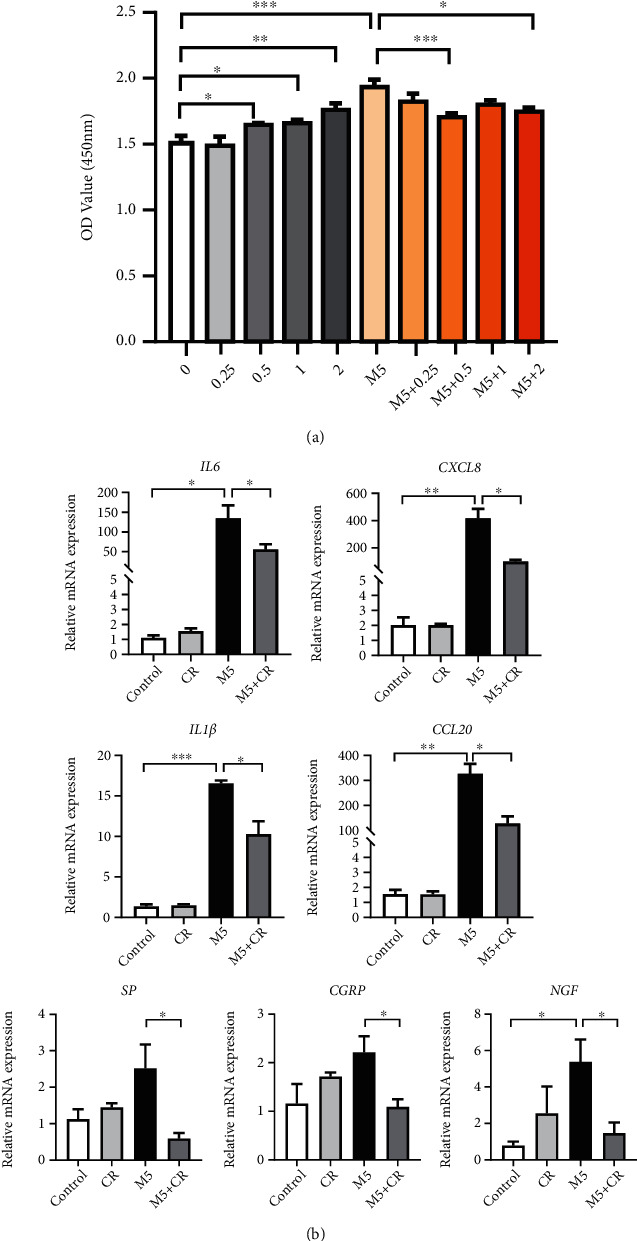
CR treatment regulated cell viability and function of M5-induced keratinocytes *in vitro*. M5 (10 ng/ml) was used to stimulate NHEKs with or without CR treatment ((a): 0, 0.25 *μ*g/ml, 0.5 *μ*g/ml, 1 *μ*g/ml, and 2 *μ*g/ml; (b): 0.5 *μ*g/ml). (a) Cell viability was measured by using CCK-8 (*n* = 4). (b) The mRNA expression of *IL-6*, *CXCL8*, *IL-1β*, *CCL20*, *SP*, *CGRP*, and *NGF* was detected by RT-qPCR (*n* = 3). The relative mRNA expression was normalized to *GAPDH* expression. Statistical analysis was performed using one-way ANOVA followed by post hoc Tukey's test. Mean ± SEM values are indicated. ^∗^*P* < 0.05,  ^∗∗^*P* < 0.01, and^∗∗∗^*P* < 0.001.

Hyperproliferative keratinocytes not only mediate inflammation but also influence itch during psoriasis [30]. Consequently, the effects of CR on keratinocytes were then investigated. The cell viability of NHEKs treated with CR was measured using CCK-8 assay. After NHEKs being treated with different concentrations of CR, no significant toxicity was observed. In contrast, 0.5, 1, and 2 *μ*g/ml of CR could slightly promote cell viability (*P* < 0.05) (Figure 6(a)). In M5 (10 ng/ml) induced inflammatory condition *in vitro*, the viability of NHEKs was significantly enhanced after 24 h (*P* < 0.001) (Figure 6(a)). However, when we combined CR (0.5 or 2 *μ*g/ml) with M5 to treat keratinocytes, the induced cell viability was inhibited (Figure 6(a)). In addition, the inflammatory cytokines and itch-related molecules were determined in NHEKs with M5 stimulation. We found that M5 (10 ng/ml) could significantly upregulate mRNA levels of *IL-6*, *CXCL8*, *IL-1β*, and *CCL20* (*P* < 0.05) (Figure 6(b)). The expression of *NGF* was also increased in the M5 group (*P* < 0.05), while *SP* and *CGRP* did not. Interestingly, CR treatment partially decreased expression of inflammatory cytokines *IL-6*, *CXCL8*, *IL-1β*, and *CCL20* and itch-related molecules *SP*, *CGRP*, and *NGF* induced by M5 (*P* < 0.05) (Figure 6(b)). In general, these results suggest that CR treatment could regulate keratinocytes viability and reduce inflammation and the expression of some itch mediators in a psoriatic cell model.

## 4. Discussion

Nowadays, most treatment protocols are targeted at the inflammatory response of psoriasis, only a few of them specifically for improving psoriasis itch. The main compositions of CR tablets were analyzed by HPLC as triptolide and epicatechin in Zhou et al.'s study in 2018 [16]. Oral administration of CR has also been successfully used in the treatment of psoriasis [19]. However, the mechanisms of topical treatment with CR in psoriasis are still unclear. Long-term oral CR always accompanies with liver, kidney, and reproductive systemic damage, so that topical preparations of CR could be a better choice preventing these side effects.

Itch is a subjective sensation, which could be quantified by observing the spontaneous behaviors of experimental animals. Rapid back-and-forth movements of the hind paw around skin lesions in mice can imitate patients' scratching behaviors. Therefore, we apply this model in our research. The results showed that scales and thickness of skin were significantly reduced in CR-treated mice, consistent with previous studies of Tripterygium wilfordii [31]. However, the erythema in the CR group was not improved than that in the IMQ group. We speculate that it may be related to irritant contact dermatitis with external application by CR. Even so, the overall inflammation of the psoriatic lesions was greatly improved, and the number of scratches was significantly reduced in the CR group. These observations suggest that CR can relieve inflammation and pruritus in psoriasis.

Excessive inflammatory response in psoriasis has been confirmed to be related with itch [2]. Several studies reported that CD4^+^ T cells were crucial for initiating and maintaining the pathogenic process of psoriasis. The percentage of CD4^+^T cells was increased in the blood of psoriasis patients [32]. CD4^+^ T cells in skins consist of different helper T (Th) cells (Th1, Th2, Th9, Th17, Th22, and Treg cells) [33]. In psoriasis, keratinocytes regulate differentiation and activation of Th17 and Th22 cells by producing IL-1*β* and IL-6 [28]. In our study, the total ratio of *αβ*T cells did not change significantly among three groups. However, the increased proportion of CD4^+^/CD8^+^*αβ*T cells in the IMQ group was decreased by topical application of CR. The ratio of CD4^+^/CD8^+^*αβ*T cells is positively related with Koebner phenomenon caused by scratching in psoriasis [34, 35]. Scratch-induced skin injury is definitely correlated with itch. Consistent with previous research, we observed an increase in neutrophil infiltration in IMQ-induced PsD in mice, as well as scratch behaviors [22, 27]. Previous studies have shown that scratch injury to epidermal keratinocytes promoted the release of CCL20 and CXCL8, which was related to the recruitment of both neutrophils and IL-17a-producing immune cells [36]. CCL20 was expressed abundantly in human psoriatic epidermis [9, 37]. Dupilumab inhibits the IL-4/IL-13 signaling pathway to reduce itching and itching in atopic dermatitis, which may reduce the release of CCL20 from keratinocytes and also reduce Th17 cell infiltration [38]. As well as in psoriasis, the enrichment of IL-17A-producing immune cells leads to produce high levels of IL-17A, which further stimulates keratinocytes producing CCL20, CXCL8, and IL-36*γ* to recruit more IL-17A-producing immune cells and neutrophils [39]. In our findings, the neutrophil infiltration and also increasing expression of *CCL20* in IMQ treated mice were both inhibited by CR. As well in vitro, dexamethasone partially inhibited scratch-induced CXCL8 and CCL20 secretion in the keratinocyte model [36]. In order to mimic a psoriatic environment, we chose an M5-induced keratinocyte model to investigate the effects of CR. As expected, CR reduced the expression levels of inflammatory cytokines *IL-6*, *IL-8*, *CXCL8*, and *CCL20*. Interestingly, we observed that CR could promote cell viability slightly in NHEKs, while in NHEKs treated with M5, CR inhibited cell viability. We speculate that CR might regulate the viability of keratinocytes under different circumstances, which should be explored in further study.

Notably, the number of mast cells was significantly reduced in lesions of CR application. Several researches observed that mast cells are hyperactivated in psoriatic lesions of progressive stage. The use of certain medications to treat psoriasis, such as glucocorticosteroids, may lead a decline of the mast cell count [11]. Our observation indicates that CR may function as an inhibitor of itch in psoriasis by decreasing the number of mast cells in skin lesions.

In addition, the itch sensation is transmitted to the brain via itch neural pathways from skin lesions [40, 41]. Sensory nerve fibers increased in the psoriatic epidermis and dermal papilla, which were stimulated (endogenous or exogenous) by various neuropeptides and neurotrophic molecule, such as CGRP, SP, and NGF. Also, in the epidermis, neuropeptides released from the nerve fibers stimulate keratinocytes to release proinflammatory cytokines such as IL-6 and IL-8. On the other hand, CGRP and SP induce the release of vasoactive amine by mast cells, promoting the infiltration of neutrophils and T cells closely related with itch [42–45]. The main source of NGF in the skin is keratinocytes [46]. The expression of NGF in psoriatic patients with pruritus is higher than that without pruritus [47]. These molecules work directly or indirectly on the nerve and lead to or aggravate the degree of itch in psoriasis [48, 49]. In our research, topical CR preparation can significantly relieve itch accompanied with reduced levels of CGRP, SP, and NGF in lesions from psoriatic mice. Additionally, in a psoriatic cell model, itch-related molecules SP, CGRP, and NGF were found decreased in NHEK cotreated with CR and M5. However, the expression of CGRP and SP in vitro was not upregulated by M5. We speculate that M5-induced keratinocytes may not be an optimal itching cell model in psoriasis.

## 5. Conclusion

**Figure 7:**
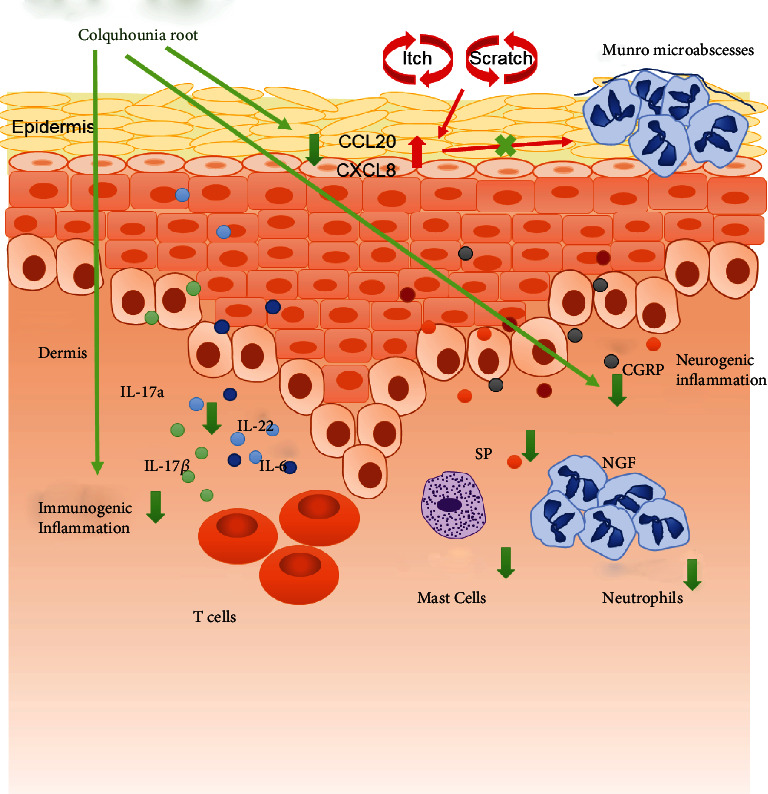
Characterization of the mechanism of CR for psoriasis pruritus improvement. CR treatment relieves the symptoms of psoriasis pruritus by inhibiting increased mast cells and neutrophils, as well as the upregulation of the inflammatory factors *IL-17a*, *IL-22*, *IL-6*, *CXCL8*, *IL-1β*, and *CCL20* and itch-related molecules *SP*, *CGRP*, and *NGF* in psoriasis.

In summary, we firstly prove that CR can not only inhibit the increased mast cells and neutrophils in lesions but also downregulate the inflammatory factors and neuropeptides and neurotrophic molecule to reduce the inflammation and itch in IMQ-induced murine model of psoriasis, which is also validated in a psoriatic cell model (Figure 7). In conclusion, CR is considered to be an ideal topical therapeutic drug for anti-itch treatment not just anti-inflammation in psoriasis.

## Figures and Tables

**Table 1:** Primer sequences for quantitative reverse-transcription polymerase chain reaction.

Gene symbol	Sequence
*Il17a*	F: 5′-TTTTCAGCAAGGAATGTGGA-3′
	R: 5′-TTCATTGTGGAGGGCAGAC-3′
*Il22*	F: 5′-ATGAGTTTTTCCCTTATGGGGAC-3′
	R: 5′-GCTGGAGTTGGACACCTCAA-3′
*Ccl20*	F: 5′-GCCTCTCGTACATACAGACGC-3′
	R: 5′-CCAGTTCTGCTTTGGATCAGG-3′
*Sp*	F: 5′-AAGCGGGATGCTGATTCCTC-3′
	R: 5′-TCTTTCGTAGTTCTGCATTGCG-3′
*Cgrp*	F: 5′-GAGGGCTCTAGCTTGGACAG-3′
	R: 5′-AAGGTGTGAAACTTGTTGAGGT-3′
*Ngf*	F: 5′-TGATCGGCGTACAGGCAGA-3′
	R: 5′-GCTGAAGTTTAGTCCAGTGGG-3′
*Gapdh*	F: 5′-AGGTCGGTGTGAACGGATTTG-3′
	R: 5′-TGTAGACCATGTAGTTGAGGTCA-3′
*IL6*	F: 5′-CCAAGAGGTGAGTGCTTCCC-3′
	R: 5′-CTGTTGTTCAGACTCTCTCCCT-3′
*CXCL8*	F: 5′-CAAGGCTGGTCCATGCTCC-3′
	R: 5′-TGCTATCACTTCCTTTCTGTTGC-3′
*IL1β*	F: 5′-GCAACTGTTCCTGAACTCAACT-3′
	R: 5′-ATCTTTTGGGGTCCGTCAACT-3′
*CCL20*	F: 5′-CTGCTACTCCACCTCTGCG-3′
	R: 5′-TTGCGCACACAGACAACTTT-3′
*SP*	F: 5′-TGATCTGAATTACTGGTCCGACT-3′
	R: 5′-TCCGGCAGTTCCTCCTTGA-3′
*CGRP*	F: 5′-ATGCAGCACCATTCAGGTCTG-3′
	R: 5′-CCAGCCGATGAGTCACACAG-3′
*NGF*	F: 5′-GGCAGACCCGCAACATTACT-3′
	R: 5′-CACCACCGACCTCGAAGTC-3′
*GAPDH*	F: 5′-CTGGGCTACACTGAGCACC-3′
	R: 5′-AAGTGGTCGTTGAGGGCAATG-3′

## Data Availability

The datasets generated during and/or analyzed during the current study are available from the corresponding author on reasonable request.

## Supplementary Materials

Supplementary Materials
*Supplementary 1* Supplementary Figure 1: gating strategy of skin cells in flow cytometry. *Supplementary 2* Supplementary Figure 2: effect of psoriatic inflammation and CR on liver and renal function of mice.
