# Supplementary material for: Topical Treatment of Colquhounia Root Relieves Skin Inflammation and Itch in Imiquimod-Induced Psoriasiform Dermatitis in Mice
Source: Mediators Inflamm. 2022 Jan 11;2022:5782922. doi: 10.1155/2022/5782922 (PMC8767412; doi:10.1155/2022/5782922)
Supplement: Supplementary Materials — Supplementary 1 Supplementary Figure 1: gating strategy of skin cells in flow cytometry. Supplementary 2 Supplementary Figure 2: effect of psoriatic inflammation and CR on liver and renal function of mice. [file 5782922.f1.docx]

**Supplementary figure 1**

**Supplementary figure 1. Gating strategy of skin cells in flow cytometry.**

**Supplementary figure 2**

**Supplementary figure 2.** **Effect of psoriatic inflammation and CR on liver and renal function of mice.** (A) Weight changing following treatment. The daily body weight of mice was compared among normal group (n=11), IMQ group (n=15) and IMQ+CR group (n=15). (B) Serum alanine aminotransferase (ALT), aspartate aminotransferase (AST), total protein (TP), albumin (ALB), blood urea nitrogen (BUN) and creatinine (CRE). All parameters were measured biochemically. n = 5 mice in three groups. Values are expressed as mean ± SEM, and analyzed by using T test. **P* < 0.05, ***P* < 0.01, ****P* < 0.001.
